# GBS-MeDIP: A protocol for parallel identification of genetic and epigenetic variation in the same reduced fraction of genomes across individuals

**DOI:** 10.1016/j.xpro.2022.101202

**Published:** 2022-03-03

**Authors:** Shiva Rezaei, Julia Uffenorde, Oliver Gimm, Mohammad Ali Hosseinpour Feizi, Stefan Miemczyk, Luiz Lehmann Coutinho, Per Jensen, Carlos Guerrero-Bosagna, Fábio Pértille

**Affiliations:** 1 Department of Animal Biology, Faculty of Natural Sciences, University of Tabriz, Tabriz 5166616471, Iran; 2 Department of Surgery and Department of Biomedical and Clinical Sciences (BKV), Linköping University, 581 83 Linköping, Sweden; 3 Avian Behavioral Genomics and Physiology Group, Department of Physics, Chemistry and Biology (IFM), Linköping University, 581 83 Linköping, Sweden; 4 Environmental Toxicology Program, Department of Organismal Biology, Uppsala University, 752 36 Uppsala, Sweden; 5 Animal Biotechnology Laboratory, Animal Science Department, University of São Paulo (USP)/ Luiz de Queiroz College of Agriculture (ESALQ), Piracicaba, São Paulo 13418-900, Brazil

**Keywords:** Cancer, Clinical Protocol, Genomics, Sequencing, High Throughput Screening, Molecular Biology

## Abstract

The GBS-MeDIP protocol combines two previously described techniques, Genotype-by-Sequencing (GBS) and Methylated-DNA-Immunoprecipitation (MeDIP). Our method allows for parallel and cost-efficient interrogation of genetic and methylomic variants in the DNA of many reduced genomes, taking advantage of the barcoding of DNA samples performed in the GBS and the subsequent creation of DNA pools, then used as an input for the MeDIP. The GBS-MeDIP is particularly suitable to identify genetic and methylomic biomarkers when resources for whole genome interrogation are lacking.

## Highlights

• Our protocol combines two well-known techniques, GBS and MeDIP.
• The GBS-MeDIP allows for parallel interrogation of genetic/methylomic variants.
• Allows for cost-efficient analysis of reduced genomes from multiple individuals.
• Ideal for the identification of genetic and/or methylomic markers at reduced cost.

## Graphical abstract

**Figure:**
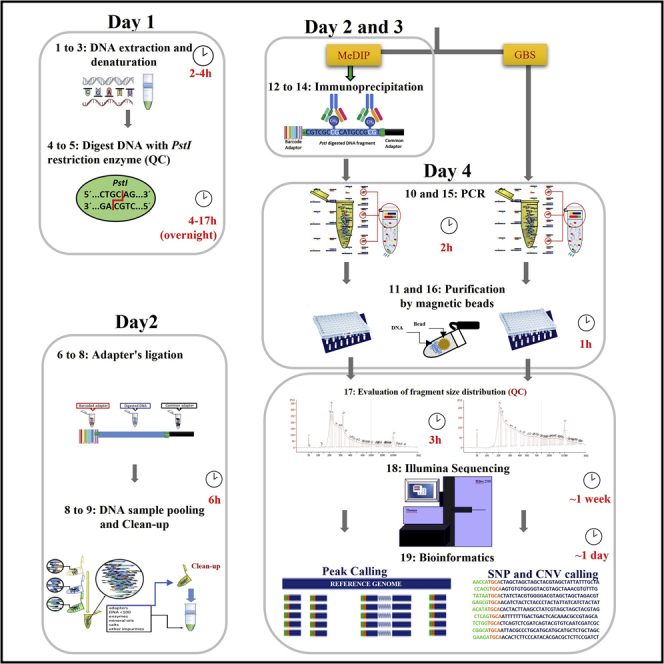

## Before you begin

The protocol below describes the specific steps using *Homo sapiens* as species model, however, we have already successfully applied this protocol using *Gallus* *gallus* (Pertille et al., 2019; Pértille et al., 2016, 2017a, 2017b, 2020)*, Canis familiaris* (Sundman et al., 2020)*, Sus scrofa* (Pértille et al., 2021), *Macrossoma macropomum* (Nunes et al., 2017, 2020), and *Mus musculus* (Unpublished data) species.

Before starting, keep in mind to clean the bench properly and to check the list of materials, reagents, and buffers needed. The library-building pipeline involves basic laboratory skills. After DNA extraction, experienced users can complete the total workflow of our optimized procedure in 4–5 days.

The method involves sequencing of the libraries in the Illumina platform, which is available at many sequencing facilities around the world. Sequencing should be performed in professional core facilities having validated instruments and experienced technicians. Bioinformatic expertise is required for later quality control and analysis of the sequencing data.

### DNA input

**Timing: 1–10 min**

1. The minimally recommended amount of individual DNA samples to be digested with *PstI* is 100 ng for successful completion of the GBS. In the present procedure, we used 400 ng of initial individual DNA material and a total of 48 samples, which yields a total of 19.2 μg.

***Note:*** The amount of DNA to be used per sample will depend on how many barcodes are available and on obtaining a final pool containing 5 μg of total DNA (quantified using Qubit Fluorometer) to be optimally used for the MeDIP.

***Note:*** The absorbance can be measured using Nanodrop to verify the purity of the library. A ratio of absorbance at 260 nm vs 280 nm of ∼1.8 is considered optimal.

**CRITICAL:** This DNA amount needs to be sufficient for the PCR amplifications of both the genomic and the methylomic libraries. We have successfully performed the PCR after using as low as 1 μg after the cleanup (step 9) of the MeDIP.

### Preparing buffers

**Timing: 0.5–4 h**

2. Before starting the protocol, make sure to have a sufficient volume of all the solutions to be used in the procedures.
  a. For the MeDIP, a 5× IP (immunoprecipitation) buffer solution and a digestion buffer (Guerrero-Bosagna and Jensen, 2015) need to be prepared (see the ‘materials and equipment’ section).
    ***Note:*** Mix well and stir the solution until no solids are visible. Apply the reagent mix on 0.2 μm syringe filter for purification and sterilization (Guerrero-Bosagna and Jensen, 2015).
  b. Prepare also a 1× IP buffer solution from the 5× IP buffer using DNase free UP water.
  c. Prepare TE buffer for oligos elution (see the ‘materials and equipment’ section.
    **CRITICAL:** All the buffers should be stored at 4°C and can be used during 6 months.

### Planning experimental sampling, barcode′s and primers′ designing

**Timing: weeks**

3. Sampling and adapters
  a. The number of individual samples included per pool should be calculated based on the yield capacity of the sequencing platform, genome size investigated, and average sequencing coverage desired per sample.

***Note:*** For example, an Illumina flowcell lane from NextSeq 550 Series can generate a maximum of 120 billion bps of sequencing yield output. Considering the Human genome (GRCh38.p13, Ensembl) size of 3,1 billion of bps, it is possible to sequence a human DNA sample with an average coverage of 38.7× (120/3.1). However, the reduced fraction of the Human genome generated by the GBS method (with the enzyme employed here) corresponds to ∼0,62 million of bps, representing approximately 2% of the whole genome. Hence, considering this reduced genome it is possible to sequence a human sample with an average coverage of 19,354.84× (120/0.0062). Therefore, considering the multiplexing capability of the GBS, and the abovementioned scenario, the GBS reduced fraction of 200 Human DNA samples can be sequenced with an average coverage of 96.77× (19,354.83/200).

**Figure 1:**
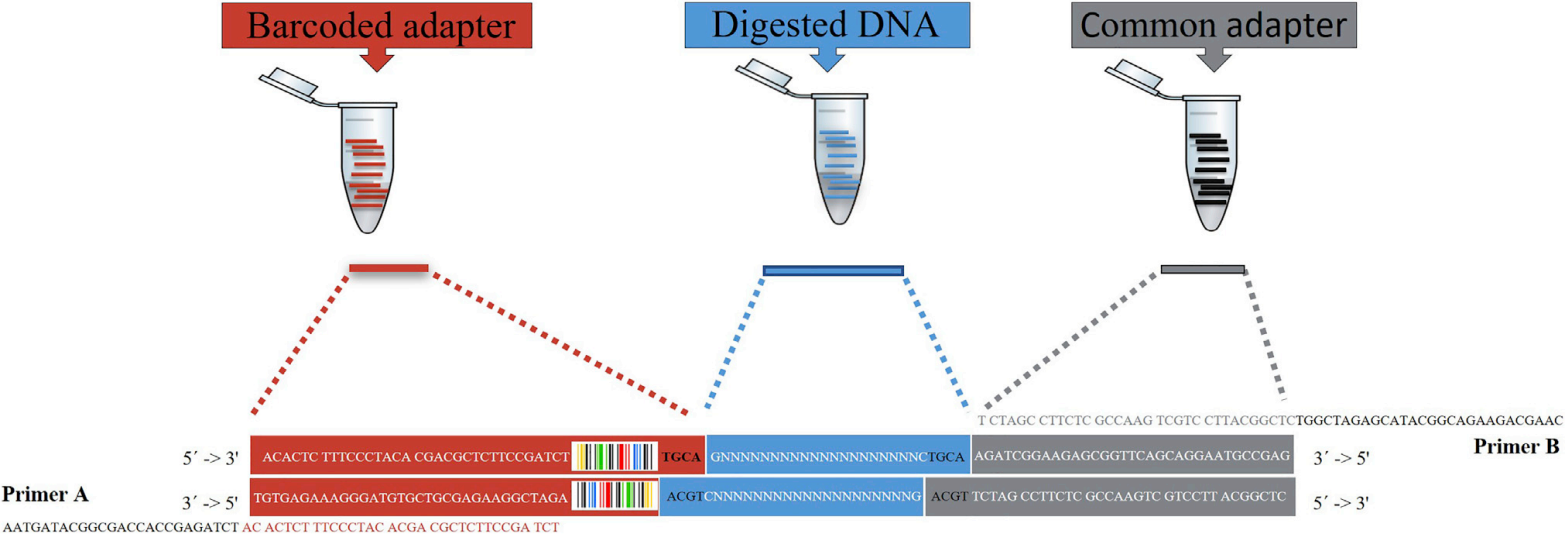
Schematic representation of the structure of each fragment bound to the common and the barcode adapters The figure represents the disposition of barcodes on the final fragment constructs after ligation, and the locations where the primers bind in order to amplify the desired fragment.

4. Designing and ordering the adapters and primers.
  a. The ‘common’ and ‘barcode’ adapters are attached to the cohesive ends produced by the *PstI* cleavage. The nucleotide sequences of these Illumina adapters are: 5′ACACTCTTTCCCTACACGACGCTCTTCCGATCTNNNNNTGCA3′ and 3′TGTGAGAAAGGGATGTGCTGCGAGAAGGCTAGANNNNN5′, where NNNNN denotes the barcode and its complementary sequences. The common adapter has a *PstI*-complementary sticky end on the 3′ end of the minus strand. The nucleotide sequences are as follow: 3′ACGTTCTAGCCTTCTCGCCAAGTCGTCCTTACGGCTC5′ and 5′AGATCGGAAGAGCGGTTCAGCAGGAATGCCGAG3’. For a schematic representation of the fragment constructs see Figure 1.
    ***Note:*** The barcode adapters contain barcodes of 5–9 bp in length on the 5′ end of the minus strand, and a 4 bp overhang on the 3′ end of the plus strand, which is complementary to the sticky ends created in the fragments after *PstI* digestion.
  b. The primers are specifically targeting both Illumina adaptors (Figure 1). PCR Primer A: AATGATACGGCGACCACCGAGATCTACACTCTTTCCCTACACGACGCTCTTCCGATCT; PCR Primer B:CAAGCAGAAGACGGCATACGAGATCGGTCTCGGCATTCCTGCTGAACCGCTCTTCCGATCT.
    ***Note:*** All adapters and primers used in this study were designed in accordance with the recommendation of the authors originally describing the GBS (Elshire et al., 2011). We have made the barcodes available in the GitHub repository https://github.com/fpertille/GBSMeDIP. To design the barcodes, we used a script written by Thoma P. Van Gurp (https://github.com/thomasvangurp/GBS_barcodes). This barcode generator designs random self-correcting barcodes that: i) do not recreate the enzyme recognition site, ii) have complementary overhangs, iii) have variable length, bases well balanced at each position, and at least 3 bp differences among barcodes to account for sequencing errors; iv) do not nest within other barcodes, and v) locate just upstream of the restriction enzyme (RE) cut-site in genomic DNA, which eliminates the need of a second Illumina sequencing (Indexing) read (Elshire et al., 2011).

### Preparing primers and barcodes

**Timing: 1–2 days**

5. Based on starting lyophilized barcodes and primers ordered in an amount of 25 nmoles, perform the following to obtain a stock solution of ∼75 nM and a working solution of ∼15 nM:
  a. dilute the lyophilized oligos in TE to 200 μM. Multiply the nmol by 5 [Volume= (nmol∗1000)/200)] to know the volume of TE to be added to the tube.
  b. For both common and barcode adapters add 25 μL of the Forward and 25 μL of Reverse oligos into 50 μL of TE solution to obtain a final volume of 100 μL with concentration of 50 μM. This is the annealing solution of adapters (see the ‘materials and equipment’ section).
  c. For the annealing of adapters, in a thermo cycler incubate at 95°C for 2 min, ramp down the temperature at −0.1°C per second to 25°C, and then incubate at 25°C for 30 min.
  d. 1st dilution:
    i. Add 6 μL of the annealed barcoded adapters from the previous step (b) and 994 μL of TE to obtain a final volume of 1,000 μL [0.3 μM]; homogenize the solution by pipetting up and down ten times.
    ii. Add 100 μL of the annealed common adapter from the previous step (b) and 900 μL of TE to obtain a final volume of 1,000 μL [5 μM].
    iii. Quantify the common and barcode adapters in Qubit™ 4 Fluorometer using a Double Strand DNA quantification kit (Cat. No. Q32851).
      ***Note:*** If after the annealing reaction there is complete annealing of the oligos, the barcode adapters should have a concentration around 6 ng/μL, while the common adapter should have a concentration around 100 ng/μL.
  e. 2nd dilution – **Stock solution**:
    i. To each new 0.2 mL tube/well add 300 ng of each barcode adapter, 300 ng of the common adapter, and add TE to obtain a final volume of 200 uL [∼75 nM]. The final concentration of DNA in this solution is 3 ng/μL (see the ‘materials and equipment’ section).
  f. 3rd dilution - **Working** **solution:**
    i. 1:4 of the stock solution prepared with TE. The final concentration of the DNA in this solution is 0.6 ng/μL [∼15 nM].

### DNA denaturation and digestion

**Timing: 15–20 min**

6. To improve the performance of the *PstI* enzymatic digestion of human genome, we added a DNA denaturation step before applying the RE digestion.

**CRITICAL:** In human samples this was a crucial step for successful digestion. We believe this process helps denaturing proteins that interfere with the activity of *PstI* in the subsequent step.

## Key resources table

**Table:** 

REAGENT or RESOURCE	SOURCE	IDENTIFIER
**Antibodies**		

5-methylcytosine (5-mC) monoclonal antibody cl. b - Classic	Diagenode	Cat#C15200006

**Chemicals, peptides, and recombinant proteins**		

100 bp DNA Ladder	Thermo Scientific	Cat#15628019
Agilent High Sensitivity DNA kit	Agilent	Cat#5067- 4626
Cellulose Acetate (CA) Membrane Syringe Filter	Thermo Scientific	Cat#722-2520
dNTP mix	New England Biolabs	Cat#N0447L
Dream Taq DNA polymerase	Thermo Scientific	Cat#EP0703
Ethanol for molecular biology^a^	Merck	Cat#64-17-5.
Glycogen	Thermo Scientific	Cat#AM9510
LightCycler® 480 SYBR Green I Master	Roche	Cat#4887352001
Nuclease-Free Ultra-Pure (UP) Water	QIAGEN	Cat#129114
Orange DNA Loading Dye 6×	Thermo Scientific	Cat#R0631
QIAGEN Proteinase K	QIAGEN	Cat#19131
Sodium chloride (NaCl) for molecular biology	Sigma-Aldrich	Cat#S67442
Sodium phosphate dibasic for molecular biology^b^	Sigma-Aldrich	Cat#S3264
Sodium phosphate monobasic for molecular biology	Sigma-Aldrich	Cat#S3139
SYBR™ Safe DNA Gel Stain^c^	Thermo Scientific	Cat#S33102
Triton X-100 for molecular biology^d^	Sigma-Aldrich	Cat#T8787
UltraPure™ 0.5 M EDTA, pH 8.0	Thermo Scientific	Cat#15575020
UltraPure™ 1 M Tris-HCI, pH 8.0^e^	Thermo Scientific	Cat#15568025
UltraPure™ SDS Solution, 10%^f^	Thermo Scientific	Cat#24730020

**Critical commercial assays**		

Agencourt AMPure XP	Beckman Coulter	Cat#A63880
Pierce™ Spin Cups – Paper Filter	Thermo Scientific	Cat#69700
Protein A/G PLUS-Agarose	Santa Cruz Biotechnology	Cat#sc-2003
*PstI* restriction enzyme	New England Biolabs	Cat#R0140S
QIAquick PCR Purification Kit	QIAGEN	Cat#28106
Qubit™ dsDNA Quantitation, high sensitivity	Thermo Fisher Scientific	Cat. No. Q32851
T4 DNA ligase	New England Biolabs	Cat#M0202L

**Oligonucleotides**		

Barcodes (see section “before you begin”)	This paper	N/A
Primers (see section “before you begin”)	This paper	N/A

**Other**		

Bioanalyzer System	Agilent	Cat#G2939BA
Centrifuge Tubes	N/A	N/A
Digital Dry Bath	AccuBlock™; Labnet	N/A
Digital Scale	N/A	N/A
Filtered Pipette Tips	N/A	N/A
Gel Documentation System	Bio-Rad	Cat#1708265
Horizontal Gel Electrophoresis System	Bio-Rad	Cat#1640301
Lab gloves	Fisher Scientific	N/A
Low protein Binding Collection Tubes	Fisher Scientific	Cat#90411
Magnetic plate	Thermo Scientific	Cat#12331D
Microcentrifuge	Thermo Scientific	Cat#75002411
Microcentrifuge tubes	Fisher Scientific	Cat#11326144
Microwave oven	NN-ST651; Panasonic	N/A
Mini centrifuges	Thermo Scientific	Cat#75004061
Mini Incubator	Labnet Mini Incubator; Labnet	N/A
Nanodrop Spectrophotometer	Thermo Scientific	Cat#ND-2000
Parafilm Laboratory Sealing Film PARAFILM®	Merck	N/A
PCR tubes	Thermo Scientific	Cat#AM12225
Pipettes	Thermo Scientific	N/A
qPCR plate	Starlab	Cat#l1402-9800
qPCR plate seal	Starlab	Cat#E2796-9795
Qubit™ 4 Fluorometer	Thermo Fisher Scientific	Cat#Q33238
Real-Time PCR Thermocycler System	Roche, LightCycler® 480 Instrument II	Cat#05015278001
Real-Time PCR White Plates	Thermo Scientific	Cat#AB0900W
Refrigerated Centrifuge	Eppendorf	N/A
Rotating Mixer	RotoBot™; Benchmark	N/A
Thermocycler	Bio-Rad	Cat#1852196
Vortex Mixer	Thermo Scientific	Cat#88880017TS

**CRITICAL:**^a^Keep in mind that ethanol is flammable

**CRITICAL:**^b^Use lab gloves and coat under a chemical fume hood to avoid inhalation, ingestion, or skin absorption of the Sodium phosphate dibasic powder.

**CRITICAL:**^c^Although safe, SYBR still needs to be carefully handled

**CRITICAL:**^d^Triton X-100 can produce severe eye irritation and burns. Use lab gloves and coat for avoiding of inhalation, ingestion, or skin absorption

**CRITICAL:**^e^UltraPure™ SDS Solution- SDS can be toxic and irritant, with a risk for severe eye damage. Use lab gloves and coat for avoiding of inhalation, ingestion, or skin absorption.

**CRITICAL:**^f^Use lab gloves and coat to avoid inhalation, ingestion, or skin absorption of the UltraPure™ 1 M Tris-HCI.

## Materials and equipment

**Table:** 5× IP Buffer

Reagent	Final concentration	Amount
Na-Phosphate 100 mM (pH 7.0)	50 mM	50 mL
NaCl 5 M	0.7 M	14 mL
Triton X-100 **∗CRITICAL**	0.25×	250 μL
DNase free UP water	n/a	35.75 mL
**Total**	**5×**	**100 mL**

**Table:** Digestion buffer

Reagent	Final concentration	Amount
Tris–HCl 1 M (pH 8.0)	50 mM	5 mL
EDTA 0.5 M (pH 8.0)	10 mM	2 mL
SDS 10%	0.5%	5 mL
DNase free UP water	n/a	88 mL
**Total**	**n/a**	**100 mL**

**Table:** TE buffer

Reagent	Final concentration	Amount
Tris–HCl 1 M (pH 7.5)	10 mM	1 mL
EDTA 0.5 M (pH 8.0)	1 mM	200 μL
DNase free UP water	n/a	98.8
**Total**	**n/a**	**100 mL**

**CRITICAL:** Store the buffers at 4°C for short-term use (maximum 6 months) or −20°C to −80°C for long-term storage.

**Table:** Annealing solution

Reagent	Final concentration	Amount
Forward Oligo 200 μM	50 μM	25 μL
Reverse oligo 200 μM	50 μM	25 μL
TE Buffer	n/a	50 μL
**Total**	50 μM	100 μL

**Table:** Stock solution of adapters

Reagent	Final concentration	Amount
barcode adapter 6 ng/μL [0.3 μM]	300 ng	50 μL
common adapter 100 ng/μL [5 μM]	300 ng	3 μL
TE Buffer	n/a	147 μL
**Total**	75 nM	200 uL

**CRITICAL:** Store the adapters (regardless of concentration) at 4°C for short-term use (maximum one week) or −20°C to −80°C for long-term storage.

## Step-by-step method details

This section lists the major steps and provides step-by-step details and timing for each major step.

### DNA extraction

**Timing: 2–4 h**

DNA samples are extracted with Qiagen DNeasy blood and tissue kit (Cat. No. 69504) or any other high-quality DNA extraction kit according to the manufacturer's protocol.

1. Follow the protocol of Qiagen Dneasy blood and tissue kit.
2. Measure the DNA concentration of the samples using Qubit fluorimeter and perform the necessary dilutions to obtain a minimum final concentration of 15 ng/μL.

**Pause point:** isolated DNA can be stored at −20°C for use in subsequent steps.

***Note:*** Run the DNA samples on 1% agarose gel electrophoresis to verify the integrity of the extracted DNA.

**CRITICAL:** It is recommended to use the Qubit™ Fluorometric DNA Quantification system (Thermo Fisher Scientific) to measure the DNA concentration, and nanodrop to check purity (a ratio of absorbance at 260 nm vs 280 nm of ∼1.8 is considered optimal). To prepare the Qubit™ reagents, follow the instruction of its kit. It is recommended to consider the results of Qubit™ measurements as the basis for calculating the amount of DNA. The concentrations measured should allow the addition of 400 ng of each individual DNA to be digested with the *PstI* enzyme within a reaction volume of 30 μl (see below of the detailed components of the *PstI* digestion reaction).

### DNA denaturation

**Timing: 15–20 min**

In order to improve the performance of the *PstI* enzymatic digestion of **human** genome samples, we added a DNA denaturation step of 95°C for 10 min followed by immersion in ice for 5 min before proceeding with the RE digestion. This step was not needed when using non-human DNA.

3. Perform DNA denaturation by incubating the samples for 10 min at 95°C. Transfer the DNA samples to ice and maintain for 5 min.

***Note:*** we recommend performing denaturation in 1.5 mL tubes

***Note:*** This step is critical when working with human samples, and optional when working with non-human.

### DNA digestion

**Timing: 4–16 h**

Next generation sequencing methodologies use enzymatically or mechanically sheared DNA. Our protocol, however, uses *PstI* restriction enzyme (RE) to fragment the DNA samples. Our approach starts with an enzymatic cleavage of the genome to produce fragments with overhangs followed by the ligation of barcodes (for posterior identification of individual samples) and Illumina adapters. Initially, we evaluated different REs, both *in silico* and *in vitro*, including *PstI*, *MseI*, *ApeKI, SbfI, MspI*, and *EcoRI* (Pértille et al., 2016). Among these enzymes tested, *PstI* was the most efficient in enriching regions of genomes of our interest (humans, chicken, pig, mouse) in a suitable length size (ranging from 200 to 500 bps) for sequencing. Importantly, *PstI* recognizes the 5′CTGCAG 3′ motif and cuts the genome in a methylation insensitive manner, as this motif does not contain CpG sites.

***Note:*** Prepare the *PstI* enzyme and its buffer as a pre-mix. The pre-mix volume should be calculated considering a larger number of samples to account for pipetting errors. Use 0.2 mL vials (tubes or plate′s wells) for the digestion reactions.

4. Prepare a digestion mix according to the indications below for each sample.

***Note:*** Use reverse pipetting due to the viscosity of the solution.

**Table:** 

Reagent (Cat#R0140S)	Amount (1×)
*PstI* (20 U/μL)	0.5 μL
10× NEB buffer	3 μL
UP Nuclease free H2O	complete to final volume of 30 μL
Extracted DNA	400 ng (calculate for each sample)
**Total**	30 μL

5. After mixing each DNA sample with the digestion reagents, immediately place the vials in a heating block. The recommended incubation time is **3** **h** for non-human DNA and **16** **h** for human DNA at **37°C,** followed by **20 min** at **85°C.**

***Note:*** Overnight incubation (16 h) with the *PstI* RE is optimal for Human samples. When using a plate, we recommend mixing by pipetting rather than vortexing. Overnight digestion can generate uneven evaporation among samples, therefore we recommend drying the samples and then resuspend them in 30 μL of UP water.

**CRITICAL:** The enzyme should be inactivated since there is no further purification step before the ligation of the adapters. The inactivation conditions of the PstI used in this protocol (20 min at 85°C) are described in step 5.

**CRITICAL:** Assess the digestion of the samples with gel electrophoresis. A homogeneous smear of fragments (QC) is expected for successful digestions. If incomplete digestion is observed in this step, the reaction needs to be optimized.

**CRITICAL:** For long-term storage, keep the plate with the remaining digested DNA at −20°C to −80°C. Troubleshooting

**Pause point:** Digested DNA can be stored at −20°C before being used in the next step.

### Adaptor ligation, sample pooling, and purification

**Timing: 6 h**

After the DNA is fully digested by the enzyme, two Illumina adapters are added at the ends of each digested DNA fragment. This is a crucial step for the clustering of the Illumina probe within the sequencing flowcell. One of the adapters is called the ‘common adapter’, which is the same for all the samples. The other is called the ‘barcode adapter’, which allows for the later interindividual identification of the sequences through bioinformatic analyses. After the ligation of the adapters, the purification step is performed on the pools and not in the individual samples.

***Note:*** A stock solution (3 ng/μL) of adapters needs to be prepared. This stock solution is further diluted 1:4 with TE water to obtain a working solution of 0.6 ng/μL. Please, look at “Before start 1–3” section for a detailed description of the adapter preparations.

***Note:*** Before starting the ligation step, register which sample will be assigned to each barcode. To avoid mistakes, it is recommended to use a multi- channel pipette for adding the adaptors. Before pipetting adaptors, vortex the containing plate with the lowest speed and then spin down the content by centrifuging the plate for 30s at low speed.

6. In a PCR plate, mix 3 μL of adapters with 8 μL of each *PstI* digested DNA samples (∼100 ng of DNA). Vortex the plate at the lowest speed (while the master mix is prepared for the next step) and spin down at around 21°C–22°C.

***Note:*** the volume of adapters needs to be optimized according to the study species. The QC for this step can only be observed after step 11. For this, 1.5% agarose gel electrophoresis is performed with 1 μl of the amplified DNA to visualize whether adapter dimers were formed (∼120 bps), which should not occur in a successful amplification. Troubleshooting

7. Prepare a Ligation Master mix according to the following table, considering a larger number of samples to account for pipetting errors. Vortex the plate and spin down for 30 s at around 21°C–22°C.

***Note:*** use reverse pipetting due to the viscosity of the solution.

***Note:*** Consider the volume of DNA and adapters added in the previous step to account for a final volume of 30 ul.

**Table:** 

Reagents (Cat#M0202L)	Amount (1×)
DNA + adapters from step 6 (0.6 ng/μL)	Up to 8 μL containing 100 ng of digested DNA + 3 μL of adapter (0.6 ng/μL)
UP Nuclease free H_2_O	complete to final volume of 30 μL
NEB Buffer 10×	3 μL
NEB T4 DNA ligase	1 μL
**Total**	30 μL

8. Incubate the ligation reaction immediately in a heat block for 2 h at 22°C followed by 30 min at 65°C.
9. Spin down and collect the whole content (30 μL) of each reaction in the PCR plate to make one pool in a 1.5- or 2-mL tube. The purification is then immediately performed using the Qiagen QIAquick PCR purification kit, as described in Box 1.
  Box 1 **PCR purification using Qiagen QIAquick kit**
  1. Calculate the final pool volume.
  2. Transfer the pool to one 15 mL falcon tube, then add 5 volumes of PB buffer and mix well using vortex.
  3. Place the DNA pool in the provided silica filter column and spin. The pool should be divided considering a maximum volume of 750 μL per column and maximum filtering capacity of 1.5 mL per column.
  4. Centrifuge for 1 min at 10,000×*g* at around 21°C–22°C.
  ∗ Discard the flow through from the collecting tube and repeat step 4, keeping the filter for the next step.
  5. Add 750 μL of PE buffer in the column (centrifuge, discard the flow through, centrifuge again and discard the flow through again to ensure that the PE buffer is completely out of the column).
  **CRITICAL:** Ensure that ethanol (96%–100%) has been added to the PE Buffer
  6. Add 30 μL of EB buffer to the center of each column
  7. Incubate for 1 min at around 21°C–22°C.
  8. Centrifuge for 1 min at 10,000×*g* and at around 21°C–22°C.
  9. Add 30 μL of EB buffer again.
  10. Incubate for 1 min at around 21°C–22°C.
  11. Centrifuge for 1 min at 10,000×*g* and at around 21°C–22°C.
  12. Mix the contents of all the tubes into one single tube.
  13. Measure the DNA concentration with fluorometer (Qubit™).
  ***Note:*** If necessary, concentrate the sample using a speed vacuum instrument at 55°C; the maximum volume of the PCR reaction for the next step should not exceed 25 μL.

***Note:*** In case the number of samples per pool is higher than 50, 30 μl of each reaction can be directly transferred to a 15 mL falcon tube.

**Pause point:** In this step, the sample may be stored in −20°C until the protocol is resumed.

**CRITICAL:** For long-term storage keep the samples at −20°C to −80°C.

### GBS-PCR (DNA/barcode ligation QC)

**Timing: 3 h**

After PCR purification of the pooled barcoded and ligated DNA samples, a PCR reaction is performed both to i) confirm the ligation of adaptors and barcodes to DNA fragments, and ii) to produce the genomic library (GBS). A recommended PCR protocol is provided bellow:

10. Prepare the PCR reaction on ice according to following reagents and immediately perform the amplification in the thermocycler according to the following conditions:

***Note:*** Optimized PCR mix reaction using the Thermo Scientific Dream Taq DNA Polymerase (EP0703)

**Table:** 

PCR reaction	Amount (1×)
Dream Taq buffer (10×)	2.5 μL
UP Nuclease free H2O	complete to 25 μL
dNTP mix (10 mM)	0.5 μL
Primer A (20 mM)	0.55 μL
Primer B (20 mM)	0.55 μL
Dream Taq enzyme (5 u/ μL)	0.375 μL
DNA from step 9	50 ng
**Total volume per PCR reaction**	**25 μL**

**Table:** 

PCR cycling conditions			
Steps	Temperature	Time	Cycles
Initial Denaturation	95°C	7 min	1
Denaturation	95°C	1 min	18 cycles
Annealing	65°C	30 s	
Extension	72°C	30 s	
Final extension	72°C	5 min	1
Hold	4°C	forever

**CRITICAL:** Keep all reagents and samples on ice before starting the PCR; for long-term storage of the PCR product, keep the samples at −20°C to −80°C.

***Note:*** The GBS-PCR product is not only a validation step for the ligation step but also generates the genomic library for sequencing after a step of PCR purification. The PCR DNA input used here should be at least 50 ng.

***Note:*** Verify the amplification of the GBS-PCR by running the PCR product and the non- template control on 1.5% agarose gel electrophoresis. Troubleshooting

11. GBS-PCR purification by using magnetic beads:
  ***Note:*** The purification step of the GBS library can be performed together with the purification of the GBS-MeDIP library in step 16. Before starting, keep the Ampure XP beads at around 21°C–22°C for at least 30 min.
  a. Vortex the PCR product and split the content across the wells (0.2 mL) of a PCR plate by adding 10 μL fractions to each well.
  b. Gently shake the magnetic beads tube (Agencourt AMPure XP, Beckman coulter (A63880)) until a homogeneous solution is observed.
  c. Add 18 μL of Agencourt AMPure XP to the samples (10 μL of PCR product) in each well and mix 10 times through pipetting.
  d. Incubate the PCR plate for 5 min at RT for maximum recovery; the color of the mix should be brown.
  e. Place the PCR plate on a magnetic bead separation rack and wait for 5 min (keep the plate and rack together until step **j**)
  f. Discard the supernatant being careful to avoid touching the beads
  g. Transfer 200 μL of ethanol 70% (vol/vol) to each well and incubate for 1 min at RT.
  h. Discard the supernatant being careful to avoid touching the beads.
  i. Repeat the process of washing (for a total of two washes).
  j. Remove the plate from the magnetic rack and place it at RT until the ethanol is evaporated.
  k. Dilute the samples in 11 μL of miliq H_2_O while mixing them through pipetting; the color of the mix should turn brown.
  l. Pool the purified GBS-PCR samples together into one well of the plate and maintain this pool for 2 min at around 21°C–22°C.
  m. Place the PCR plate again in the magnetic rack for 2 min in order to separate the beads from the reaction.
  n. Transfer 20 μL of the supernatant to a sterile tube.
  o. Measure the concentration of the sample with Qubit™.
    ***Note:*** QC: Verify the quality of the library by running 1 μl of purified DNA on a bioanalyzer device and/or by performing electrophoresis on 1.5 % agarose gel.
    **Pause point:** Store the purified sample at −20°C or −80°C until they are sent for NGS.

### MEDIP from GBS libraries

**Timing: 2 days**

The remainder GBS genomic pool (from which a fraction was used for the amplification of the GBS-PCR) is now subjected to MeDIP. The MeDIP procedure is performed according to a previously published optimization of the method (Guerrero-Bosagna and Jensen, 2015).

***Note:*** keep extra 400 ng of DNA for the GBS library to be used in case of failures during the GBS PCR or purification procedures.

### Day 1

**Timing: 1 h plus around 16 h**

12. Incubation with anti-methyl cytosine antibody
  a. Add TE buffer to the DNA pool (∼5 μg recommended) from step 9 after purification protocol (Box 1), in a final volume of 400 μL in a 1.5 mL tube. In case the volume exceeds 400 μL, dry the sample using speed vacuum.
  b. Heat-denature the DNA for 10 min at 95°C and then place it on ice for 5 min.
  c. Add 100 μL of 5× IP buffer.
  d. Add 5 μL of anti-methyl cytosine antibody (2 μg/μL).
  e. Incubate for 16 h at 4°C under constant rotation.
    **CRITICAL:** Make sure the tubes are properly sealed.
    ***Note:*** Seal the tube lid tightly with parafilm to avoid leaking.

### Day 2

**Timing: 4 h + 2 h (6 h)**

13. Binding of agarose beads (Protein A/G PLUS-Agarose, Cat#sc-2003) to DNA
  ***Note:*** use low protein binding collection tubes to minimize agarose G-protein loss.
  a. Vortex the tube of agarose beads until the solution is uniform; then quickly, before the beads sink again, add 160 μL of the agarose beads to an empty 1.5 mL tube.
  b. Centrifuge this tube for 2 min at 4500×*g* and 4°C; discard the supernatant.
  c. Transfer 500 μL of DNA mixture sample from step (12) to the tube with the concentrated agarose beads.
  d. Incubate for 2 h under constant rotation at 4°C.
    **CRITICAL:** Make sure the tubes are properly sealed.
    ***Note:*** Seal the tube lid tightly with parafilm to avoid leaking.
  e. Centrifuge the tube for 2 min at 4500×*g* and 4°C; discard the supernatant.
  f. Add 1 mL of 1× IP buffer.
  g. Incubate for 5 min under constant rotation at 4°C.
    **CRITICAL:** Make sure the tubes are properly sealed
    ***Note:*** Seal the tube lid tightly with parafilm to avoid leaking.
  h. Centrifuge for 2 min at 4500×*g* and 4°C; discard the supernatant.
  i. Repeat the steps “f, g and h” once (for a total of two washes).
    **CRITICAL:** The number of washes is critical for a successful procedure.
  j. Add 210 μL of digestion buffer to re-suspend the beads.
    ***Note:*** Because SDS precipitates when stored at 4°C, it is recommended that the solution is re-solubilized by warming it at 37°C for a few minutes, until the solution appears homogeneous. Avoid shaking the bottle of digestion buffer vigorously. Doing so will produce foam.
  k. Add 20 μL of proteinase K (20 mg/mL).
  l. Add 23 μL of DTT 0.1 M.
  m. Seal the tube and incubate for 2 h on a rotating platform at 55°C.
    **CRITICAL:** Make sure the tubes are properly sealed
    ***Note:*** Seal the tube lid tightly with parafilm to avoid leaking.

14. Purification of the sample
  a. Transfer the sample from the last step to a Pierce spin-filtering column (Pierce™ Spin Cups, Cat#69700) placed in a 1.5 mL empty tube and centrifuge at max speed for 30 s.
  b. Discard the column and keep the flow through.
  c. Add 3 μL glycogen (5 mg/mL) to the tube containing the flow through and vortex.
  d. Add 20 μL of 5 M NaCl and 750 μL of absolute ethanol and mix well.
    ***Note:*** Use ice cold reagents to improve the efficiency of the reaction.
  e. Precipitate the DNA by maintaining the tube on ice for 30 min.
  f. Centrifuge for 30 min at maximum speed (∼11,000×*g*) and 4°C, and remove the supernatant.
    **CRITICAL:** To prevent the pellet disturbance, carefully discard the supernatant.
  g. Add 1.5 mL of ethanol 70% to the pellet.
  h. Incubate for 5 min on a rotating platform at 4°C.
  i. Centrifuge for 10–15 min at 11,000×*g* and 4°C and carefully remove the supernatant.
  j. Add 1.5 mL of 70% ethanol.
  k. Incubate for 5 min on the rotating platform at 4°C.
  l. Centrifuge for 15 min at 11,000×*g* and 4°C and carefully discard the supernatant.
  m. Place the vial in a heating block at 50°C for 5 min to dry the sample.
  n. Add 32 μL of UP H_2_O and mix well.
  o. Incubate for 5 min in a heating block at 50°C.
  p. Measure the concentration of the DNA sample using Nanodrop.
    ***Note:*** After the DNA is denatured, the antibody binds to single stranded methylated DNA fragments. Because now most of the DNA will be single-stranded (Ruike et al., 2010), measuring the DNA using Qubit™ DS kit will underestimate the real concentration value.
    **Pause point:** MeDIP samples can be maintained at −20°C until further utilization in the PCR reaction.
    Troubleshooting

### GBS-MeDIP-PCR reaction

**Timing: 1.5 h**

The MeDIP captured genomic fraction, which is enriched for DNA methylation, is then PCR amplified. However, only DNA regions containing both adapters are able to be amplified and subsequently sequenced (Poland and Rife, 2012). The GBS-MeDIP-PCR is performed using 50 ng of the barcoded and immuno-precipitated (MeDIP) DNA pool as the template (obtained from step 14).

15. Prepare the PCR reaction on ice according to following reagents and immediately perform the amplification in the thermocycler according to the following conditions:

***Note:*** There is no specific reason for using the polymerase enzyme specified here.

**Table:** 

PCR reaction	1×
Dream Taq buffer (10×)	2.5 μL
UP Nuclease free H_2_O	complete to 25 μL
dNTP mix (10 mM)	0.5 μL
Primer A (20 mM)	0.55 μL
Primer B (20 mM)	0.55 μL
Dream Taq enzyme (5 u/ μL)	0.375 μL
DNA from step 14p	50 ng
**Total volume per a PCR reaction**	**25 μL**

**Table:** 

PCR cycling conditions			
Steps	Temperature	Time	Cycles
Initial Denaturation	95°C	7 min	1
Denaturation	95°C	1 min	23 cycles
Annealing	65°C	30 s	
Extension	72°C	30 s	
Final extension	72°C	5 min	1
Hold	4°C	forever

**CRITICAL:** Keep all reagents and samples on ice before starting the PCR; for long-term storage of the PCR product, keep the samples at −20°C to −80°C.

**CRITICAL:** Load 1 μL of the PCR products on a 1.5% agarose gel and perform electrophoresis to confirm amplification of the expected smear of fragments containing adaptors. Troubleshooting

### Purification of PCR products and fragment length assessment

**Timing: 2 h**

16. Perform PCR purification with magnetic beads as previously mentioned in step 11 (GBS-PCR purification with magnetic beads).

**CRITICAL:** Measure the sample concentration with Qubit. The expected concentration is 3–5 ng/μl.

**Pause point:** Store the purified sample at −20°C until NGS is performed.

### Evaluation of fragment size distribution (QC)

**Timing: 3 h**

In this step, the sizes of the library fragments are assessed through a fragment analyzer equipment.

17. Run 1 μL of the final purified PCR products from each produced library (GBS and GBS-MeDIP) on a bioanalyzer following the manufacturer′s protocol.

***Note:*** We use the Bioanalyzer (Agilent Bioanalyzer 2100, Invitrogen) with the Agilent High Sensitivity DNA kit (Catalog numb. 5067-4626).

***Note:*** Both the GBS and GBS-MeDIP libraries are now ready to be sequenced on different lanes using the Illumina platform.

### Next generation sequencing

**Timing: 1 week**

The libraries obtained after amplification and purification are quantified and paired-end sequenced (125 cycles) using the Illumina platform.

18. HiSeq SBS V4 kit is used in the HiSeq2500 Illumina platform following the fabricant pipelines.
  ***Note:*** More recently, we have also successfully sequenced our libraries in the NovaSeq sequencer, also from Illumina. Our sequencing is performed at the SNP&SEQ facilities of SciLifeLab (Sweden).

### Data processing and alignment

**Timing: 1 day**

After sequence, the facility makes the data available in .fastq format

19. The bioinformatic pipelines applied to the data obtained from the sequencing facility have been previously reported (Pértille et al., 2017a; Pertille et al., 2019; Guerrero-Bosagna et al., 2020; Pértille et al., 2020, 2021).

***Note:*** A barcoding system is originally used in the GBS method (Poland and Rife, 2012), and also in the GBS-MeDIP, which involves the addition of a barcode adapter (in order to identify each one of the individual samples) and a common adapter (suitable for Illumina sequencing) to the individual genomic fragments produced by the *PstI* digestion. Because of this, the methylation and genomic signals associated to each individual sample can be distinguished after the sequencing process.

***Note:*** The time spent in data processing is variable and dependents on computational processing power and operator skills. Basic processing of data can be performed in one day (see the ‘data and code availability’ section for a stand-alone pipeline, and bioinformatics method description), while subsequent data analysis depends on the hypotheses to be answered and the contrasts to be compared.

## Expected outcomes

### First quality control (QC) step

**Figure 2:**
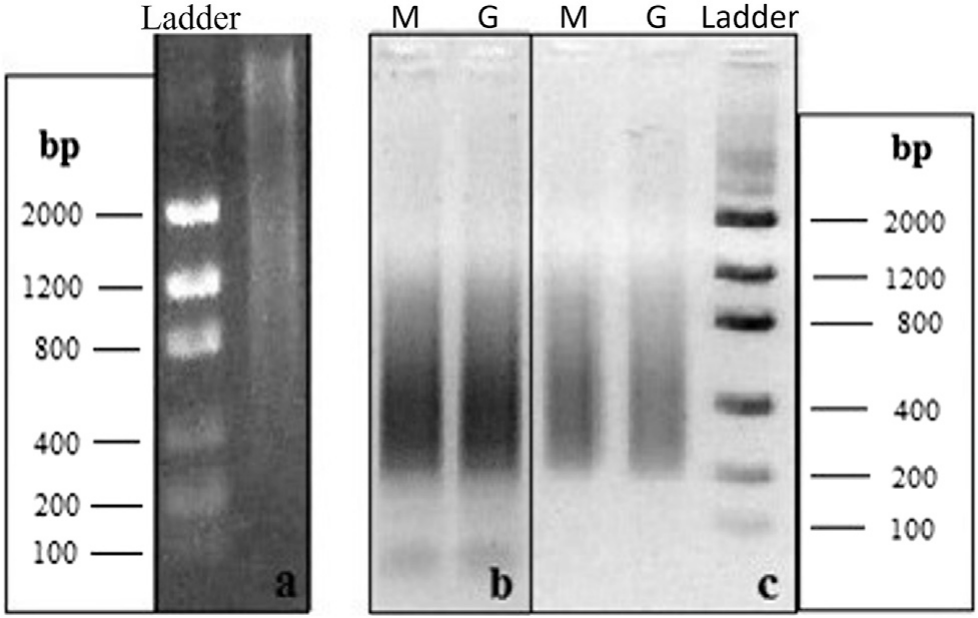
Agarose gel electrophoresis showing genomic digestion and PCR amplification of the libraries (A–C) The figure represents the agarose gel electrophoresis of the PstI digestion reaction after 16 h PstI digestion (A); PCR amplifications of the genomic (G) and methylomic (M) libraries before (B) and after (C) purification by AMPure XP magnetic beads (representation of two pools of 24 samples each), which eliminates small fragments, including unbound adapters and primer-dimers.

This step is performed to verify the quality of the digestion reaction and the generation of suitable DNA fragment sizes for Illumina sequencing. For this, 1 μL of each digested DNA sample is run on agarose gel (1.5%) electrophoresis (Figure 2A) and the *PstI* RE digestion products are checked on agarose gel electrophoresis. The expected outcome from this step is a homogeneous gel smear. Incomplete digestion is an unwanted outcome for this step and is represented by bands usually at the top of the gel. In case of partial digestion, it is essential to optimize the digestion reaction to obtain optimal fragmentation results for all samples.

### Second QC step

#### GBS-PCR: Test the efficiency of the DNA/barcodes ligation by GBS-PCR

A PCR reaction using specific primers targeting both Illumina adaptors (Figure 1) is performed to examine the efficacy of adaptor ligation. This is a quality validation of the ligation reaction because the PCR will only amplify DNA fragments successfully ligated to the Illumina adapters in the previous step. A successful ligation to both adapters will be the template for the amplification of fragments that can be detected in agarose gel electrophoresis. The expected outcome for this step is a smear concentrated in a region corresponding to 400 bps (±200 bps) on average (Figure 2B). This PCR product of the entire *PstI* reduced genomic fraction is then purified with magnetic beads (Figure 2C) before being sent for NGS. The PCR purification is an essential step that needs to be carefully performed in order to obtain a pure library, which minimizes the presence of primer-dimers and other small fragments. A high-efficiency purification approach based on solid-phase reversible immobilization magnetic beads from the AMPure XP kit (Beckman coulter (A63880) is employed to clean the PCR products (Quail et al., 2012) generating both the genomic and methylomic libraries. The expected outcome after the purification step is the elimination of any fragment below 200 bps of length, which corresponds to unbound adapters and primer-dimers, as can be seen at Figure 2C.

### Third QC step

#### Choose the appropriate number of PCR cycles and conditions of the PCR program

The PCR is performed using taq polymerase from Thermo Fisher Scientific (EP0703). The PCR reaction mix is prepared according to the manufacturer's protocol. The temperatures, and number and length of cycles in the PCR reaction were adjusted in order to avoid primer dimers and generate unbiased amplification of the fragment constructs. In order to perform the PCR reaction, a suitable number of PCR cycles was first identified with the aim of obtaining a minimal amount of dimeric sub-products.

**Figure 3:**
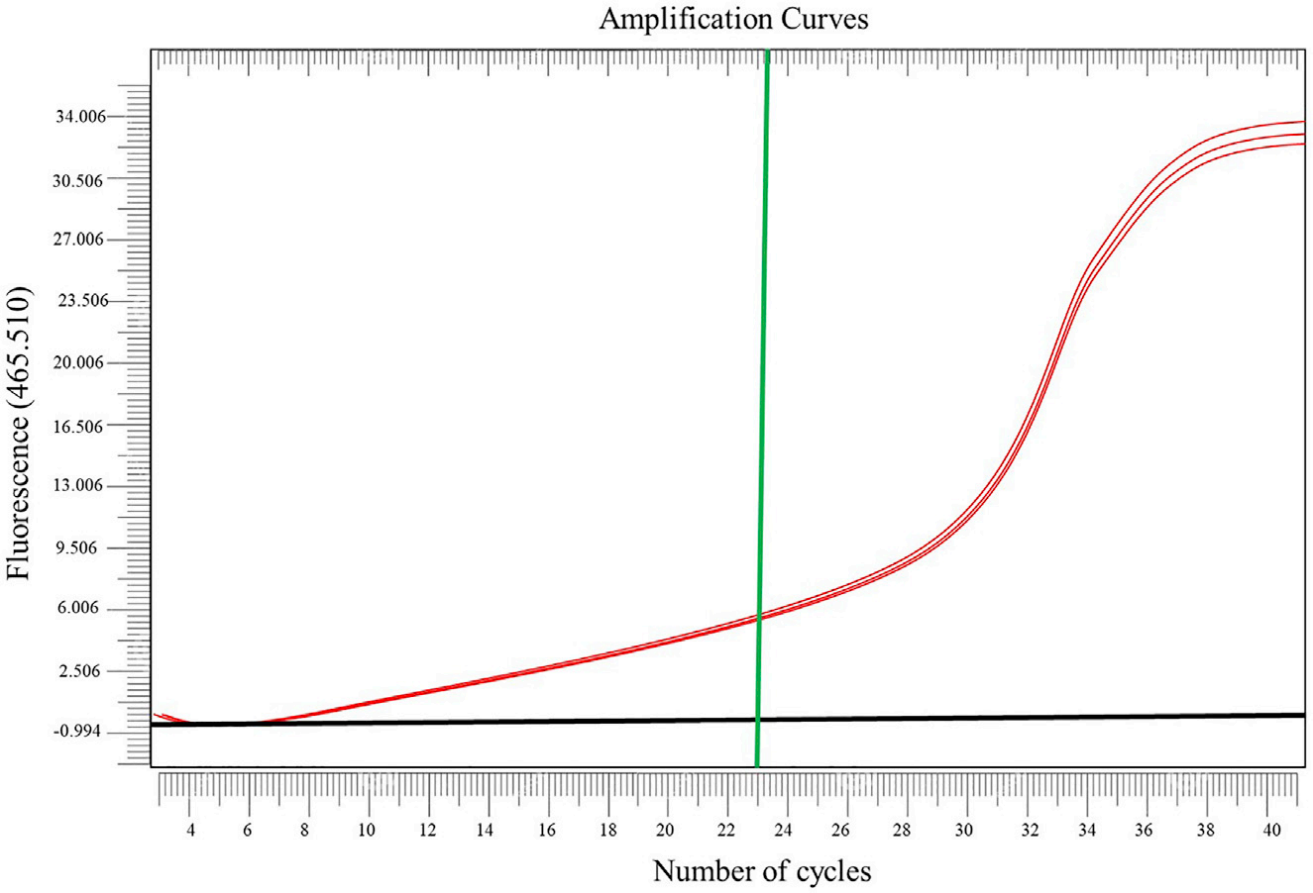
Real time amplification curve of GBS MeDIP-PCR Green line represents the number of cycles chosen; red lines represent the qPCR amplification curve of the library in triplicate, and the black line is the Ct threshold, which is the point where fluorescence of the PCR product can be detected above the background signal. The x and y axes represent number of cycles and fluorescence, respectively.

For this, real-time quantitative PCR amplification curves were monitored. The end of the early non-exponential phase was considered of interest, as it represents the maximum amplification obtained where the PCR product is still on a linear increase in relation to the input. Based on this, 23 cycles are considered as the suitable number of PCR cycles for performing the amplification of the libraries produced with the GBS-MeDIP method on the human samples used in the present study (Figure 3). In case of a PCR optimization for a combination of enzyme and species different from those used in this study, this quality control optimization should be carried out and the ideal PCR cycle number should be identified.

### Fourth QC step

#### Assess the GBS-MeDIP library quality and quantity

**Figure 4:**
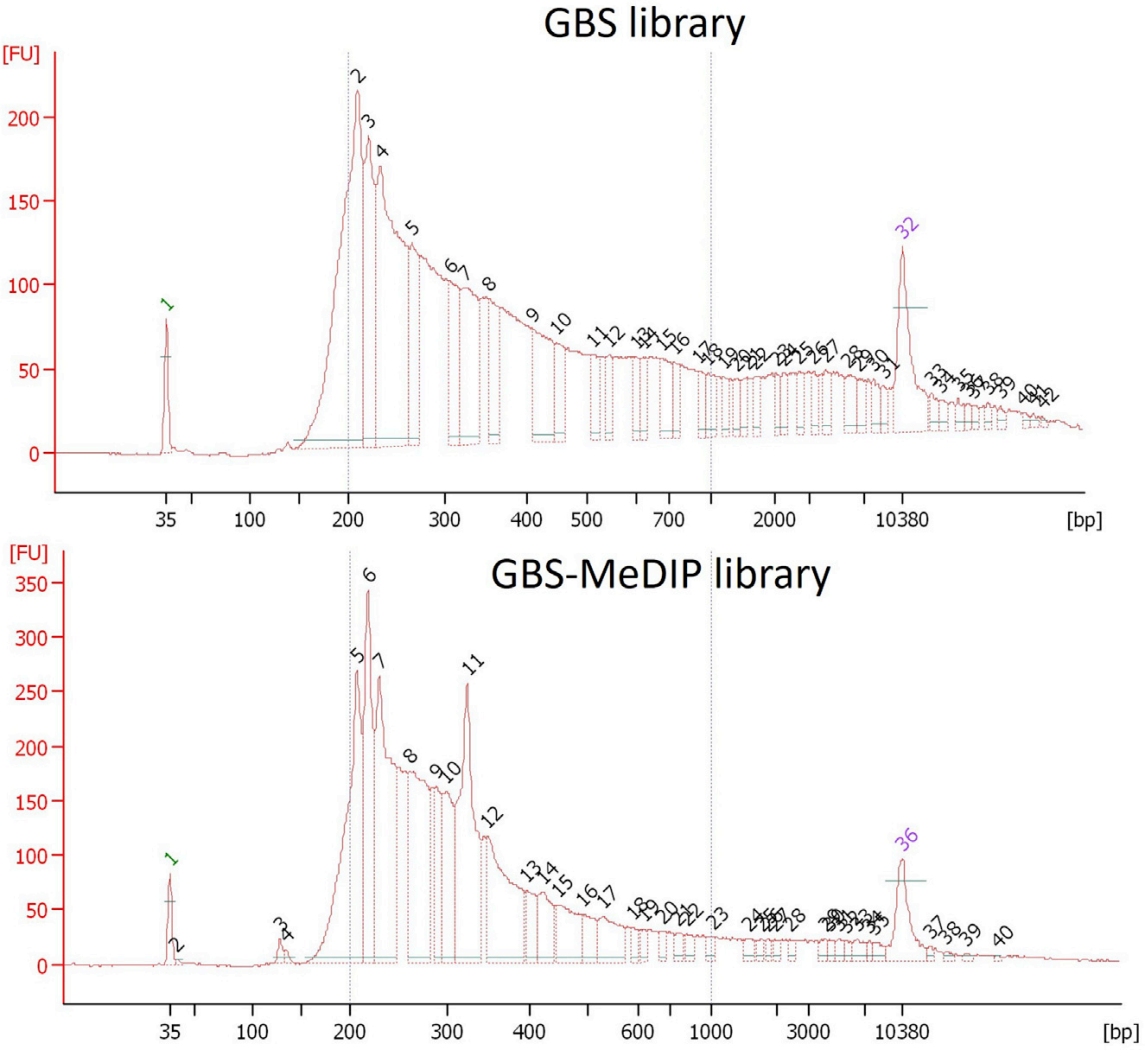
High-sensitivity chip electropherogram of the purified libraries after PCR The fragments pattern after GBS and GBS-MeDIP PCR purifications using AMpure XP magnetic beads. Green and purple lines show lower and upper markers, respectively. X and Y axes demonstrate the migration time (s) and fluorescence units (FU), respectively. Each number represents one fragment length in bps.

In this step, the sizes of the library fragments are assessed through Bioanalyzer (Agilent Bioanalyzer 2100, Invitrogen), using the Agilent High Sensitivity DNA kit (Catalog numb. 5067-4626). One microliter of the final purified PCR product is sufficient to determine whether the final amplification of the libraries is successful (Figure 4). The expected outcome is a high concentration of the fragments ranging from 200- 600 bps, and as few fragments as possible below 200 bps.

## Limitations

In general, there are two main limitations of our method. One is the small number of CpGs interrogated relative to those present in the whole genome (reduced representation). The second is that the method does not allow to analyze the methylation status of individual CpGs within the same sequenced fragment. This is primarily because the immunoprecipitated fragments have been previously restricted with the *PstI* enzyme, resulting in that the methylation signal emerging from the binding of reads is of the same length across a specific region. Because these reads contain the same number of CpGs, all CpGs within a read will associate with the same signal. Thus, the representation of DNA methylation is based on fragment rather than on individual CpGs, unless a read contains only one CpG. However, the objective of our method is not to identify individual differentially methylated CpGs, rather scanning the genome for regions that will subsequently be validated by methodologies that investigate CpG methylation at single -nucleotide resolution (e.g., conventional bisulfite sequencing).

## Troubleshooting

### Problem 1

Step 5 – incomplete digestion

### Possible reason

Samples have incorrect concentration measurements, or inaccuracy in the preparation of the enzyme mix.

### Potential solution

Re-measure the DNA concentration with Qubit™ and make sure to use the same amount of input DNA for all your samples. Prepare the *PstI* and its buffer as a premix (according to the protocol) and mix them with DNA samples by pipetting well.

### Problem 2

Step 6 –Adapter’s dimers

### Possible reason

Excess of adapters in the ligation step can enhance adapter dimer or the ligation of the adapters with very short and/or unspecific DNA fragments. Fragments from 128 to 132 bps (varying with the barcode length) are unwanted adapter dimers that can occur after ligation reaction and will hardly disappear after PCR purification using Ampure XP beads.

### Potential solution

Optimizing the amount of adapters for the DNA template is critical to reduce adapter dimers in the library and consequently to increase the sequencing yield. This procedure can be performed with a pool of the original samples. After pooling and Qiaquick PCR purification, a minimum amount of 50 ng of DNA is required as input in the PCR reaction to later check the amplification of the library and the absence of adapters dimers.

### Problem 3

Step 10 – primer dimers overshadowing the library

### Possible reason

Excess primers during the electrophoresis can mask library fluorescence on the transilluminator. Fragments of 53 bps (PF:25+PR:28) length are primer dimers that can occur after PCR reaction and should disappear after PCR purification using Ampure XP beads.

### Potential solution

Optimize the amount of primer for DNA template to be amplified in your PCR reaction to reduce primer overrun after the reaction.

### Problem 4

Step 14 – low or no concentration measured after the MeDIP

### Possible reason

The DNA pellet might have been lost during ethanol precipitation.

### Potential solution

Make sure to centrifuge the tube with the lid folds placed outwards, and pipet out the content placing the tip in the opposite side of the tube.

### Problem 5

Step 15 –weak or no GBS MeDIP- PCR

### Possible reason

The presence of PCR inhibitor elements in the sample, such as ethanol or salt.

### Potential solution

Take measures to avoid contamination of the recovered DNA after DNA precipitation; For example, carefully pipet out all the remaining ethanol left after the washing steps. Moreover, re-do the ethanol precipitation/purification to remove Na_2_Cl excess. Also, remember to keep the ethanol and NaCl solutions on ice before using them for DNA precipitation.

## Resource availability

### Lead contact

Further information and requests for resources and reagents should be directed to and will be fulfilled by the lead contacts, Fábio Pértille (fabio.pertille@ebc.uu.se) and/or Carlos Guerrero Bosagna (carlos.guerrero.bosagna@ebc.uu.se).

### Materials availability

This study did not generate new unique reagents.

## Data Availability

The acession number for the sequencing data generated with the samples reported in this paper is PRJEB35669 (www.ebi.ac.uk/ena/data/view/PRJEB35669). All the programs and R packages used in this protocol are open-source and all the scripts are available online in the manual of the programs or packages used. For more details, refer to our previous publications employing the GBS-MeDIP (Pértille et al., 2017a; Pertille et al., 2019; Guerrero-Bosagna et al., 2020; Pértille et al., 2020, 2021). We are also providing an overview of the GBS-MeDIP protocol development, a stand-alone pipeline, and bioinformatics method description in the GitHub link https://github.com/fpertille/GBSMeDIP.git
